# A review of estimating population exposure to sea-level rise and the relevance for migration

**DOI:** 10.1088/1748-9326/abb398

**Published:** 2020-11-27

**Authors:** Celia McMichael, Shouro Dasgupta, Sonja Ayeb-Karlsson, Ilan Kelman

**Affiliations:** 1 University of Melbourne, Australia; 2 Fondazione CMCC and Università Ca’ Foscari Venezia, Italy; 3 University of Sussex, United Kingdom; 4 United Nations University – Institute for Environment and Human Security (UNU-EHS), Germany; 5 Corresponding author: University College London (UCL), United Kingdom; 6 University of Agder, Norway; ilan_kelman@hotmail.com

**Keywords:** adaptation, climate change, floods, migration, sea-level rise

## Abstract

This review analyses global or near-global estimates of population exposure to sea-level rise (SLR) and related hazards, followed by critically examining subsequent estimates of population migration due to this exposure. Our review identified 33 publications that provide global or near-global estimates of population exposure to SLR and associated hazards. They fall into three main categories of exposure, based on definitions in the publications: (i) the population impacted by specified levels of SLR; (ii) the number of people living in floodplains that are subject to coastal flood events with a specific return period; and (iii) the population living in low-elevation coastal zones. Twenty of these 33 publications discuss connections between population migration and SLR. In our analysis of the exposure and migration data, we consider datasets, analytical methods, and the challenges of estimating exposure to SLR followed by potential human migration. We underscore the complex connections among SLR, exposure to its impacts, and migration. Human mobility to and from coastal areas is shaped by diverse socioeconomic, demographic, institutional, and political factors; there may be ‘trapped’ populations as well as those who prefer not to move for social, cultural, and political reasons; and migration can be delayed or forestalled through other adaptive measures. While global estimates of exposed and potentially migrating populations highlight the significant threats of SLR for populations living in low-lying areas at or near coastlines, further research is needed to understand the interactions among localised SLR and related hazards, social and political contexts, adaptation possibilities, and potential migration and (im)mobility decision-making.

## 1. Introduction: why concerns about population exposure to sea-level rise?

Between 1902 and 2015, global mean sea-level rise (GMSLR) was 0.12–0.21 m according to the Intergovernmental Panel on Climate Change (IPCC 2019, p 334). Relative to 1986–2005, additional sea-level rise (SLR) of 0.43–0.84 m is projected by 2100 (0.29–1.10 m, likely range) (IPCC 2019, p 324), although it depends particularly on the rate of Greenland and Antarctic ice sheet melting and so it could be much higher (Thomas and Lin 2020). At the moment, climate change causes SLR predominantly through sea water expanding as it absorbs heat from the atmosphere (thermal expansion) and melting of land-based snow and ice (such as from glaciers). SLR interacts with other climatic factors, such as intensifying storms and wave action with consequences already observed including infrastructure damage, coastal erosion, salination of freshwater, and land habitat loss (IPCC 2018, 2019). Future SLR is projected to affect human health and wellbeing, cultural and natural heritage, freshwater, biodiversity, agriculture, and fisheries (WHO 2018, IPCC 2018, NRC 2019).

Consequently, various attempts have been made at the global scale to assess populations exposed to SLR (Muis *et al* 2017). While these assessments use different definitions, approaches and scenarios, they seek to estimate the number of people who might be directly affected by SLR and related impacts, which defines ‘exposure’. The studies variously combine datasets on SLR, subsidence, coastal extreme weather, land elevation, population distribution, land-surface characteristics, adaptation options, and socio-economic change scenarios. One of the first global vulnerability assessments estimated that 200–250 million people per year (in 1990) were exposed to coastal flooding, defined as living below the 1-in-1000-year extreme sea level, and hence 1 m of GMSLR would increase exposure by 50% assuming no other changes (Hoozemans *et al* 1993). Recently, IPCC (2019) estimated that 680 million people currently live in the low-lying coastal zone and projected this number to reach more than one billion by 2050.

Despite the possibility for various forms of adaptation to SLR, with many people and populations already planning for adaptation *in situ* (e.g. Yamamoto and Esteban 2014), human mobility has been widely positioned as a deterministic certainty whereby climatic and environmental hazards such as SLR force people away from their coastal homes (c.f. Myers 2001). For example, Strauss *et al* (2015) state that future carbon emissions ‘*will determine which areas we can continue to occupy or may have to abandon*’ (Strauss *et al* 2015, p 13 508). The first IPCC assessment report (IPCC 1990) estimated that half a million people in archipelago and island countries might live in sites that were at risk of submergence or loss of land by 2100, contributing to increased numbers of so-called ‘*climate refugees*’ (Dronkers *et al* 1990, but see also Lewis 1990a &, 1990b). More recently, Nicholls *et al* (2011) estimated that if 2 m of GMSLR is realised by 2100, a risk of ‘*forced displacement*’ exists of up to 187 million people. Studies draw on estimates of exposure to SLR and other related hazards (e.g. living in a 1-in-100-year flood plain) as proxy indicators for population migration and relocation. In some sites, specifically low-lying coral atoll nations, population displacement is stated as being likely as even modest SLR is assumed to disrupt livelihoods and render land uninhabitable (Mcleman 2018).

Studies that estimate and forecast global and near-global exposure to, or presumed migration due to, SLR make choices about parameters, such as level of GMSLR, return periods of flooding, and time horizons. They rely on global datasets and draw conclusions on a global scale. While they provide important estimates of the scale of potential exposure, there are implicit assumptions that global trends are of primary significance for understanding the situation facing people and for planning for adaptation, so that the specificities of local sociocultural and environmental contexts are often obscured. Indeed, many analyses end at the point where local knock-on impacts and decision-making begin, irrespective of global trends. For example, the geographic distribution of coastal flooding is mapped against gridded global data sets, but the impact of this flooding on freshwater supplies for particular coastal sites is not clear and can sometimes be countered by local measures (Yang *et al* 2019).

To indicate the advantages and limitations of the studies available, and to better direct future work in this area, this paper reviews and discusses datasets and analytical methods for estimating global or near-global population exposure to SLR, with a specific focus on suggestions about SLR-related population mobility attributed to SLR and associated impacts.

## 2. Method

Three databases—Web of Science, Scopus, and Google Scholar—were searched for publications published up until April 2020 that provide quantitative estimates of population exposure to SLR and associated hazards. The full-text search string used was English only: population* AND coast* AND flood* AND ‘sea level*’ AND (global OR international* OR worldwide) AND (model* OR indic*) followed by snowball sampling of citations in publications found. The selected publications were restricted to those published in English and with a focus on global or near-global estimates. Publications with a regional or smaller-scale focus (c.f. Ericson *et al* 2006, Dasgupta *et al* 2009, 2011, Hinkel *et al* 2011, Anderson *et al* 2018, Taherkhani *et al* 2020) were therefore excluded. Provided that the publication appeared in the database search or through the snowballing, it was considered irrespective of being peer-reviewed or not, but wider searches were not conducted to capture all the grey literature on that rationale that the wider material had not necessarily been validated through scientific investigation. No time limit was placed on the searches, but the selected publications range from 1993 to 2019. From the results, manual screening was completed based on the authors’ expertise to seek comparability among the studies while being strict about the scoping of and definitions used by this review.

Thirty-three publications met the inclusion criteria: 30 peer reviewed journal articles, 1 book, 1 working paper and 1 report (table 1). The final study selection of 33 publications resulted in 11 publications estimating population exposure to specific levels of GMSLR, 13 publications estimating populations living in coastal floodplains, and 12 publications estimating populations living in LECZ or near-coastal zones. These publications were analysed by extracting the key information shown in the columns of table 1 permitting a synthesis of what is known about this review’s research topic of estimating population exposure to SLR and the relevance for migration. To fulfil this review’s mandate, the columns in table 1 focus on the publications’ aims, sources of information (data sets) and analysis methods, time frames considered for forecasts, scenarios and numbers for population exposure, any assumptions or implications related to migration, and the data or analysis challenges mentioned, thereby also permitting discussion in the next sections of this review regarding what is not mentioned in these publications.

**Table 1.** Global or near-global studies of population exposure to GMSLR and/or populations living in low elevation ^a^ coastal zones (LECZs) and coastal floodplains.

	Key data sets	Aims	Time-Frame	Exposed population	Estimate/Key finding	Migration
**Estimates of population exposure to specified levels of SLR**						
Nicholls *et al* 1999	• Global SLR scenarios (Hadley Centre) • Rise in atmospheric carbon dioxide concentration from 354 ppmv (1990) to 731 ppmv (2080s) • World Bank 1994/95 global population with estimates to 2150 • (GDP) from Energy Modelling Forum 14 GDP/capita scenario	To estimate flooding due to storm surges, and wetland losses due to SLR.	2020s, 2050s, 2080s	People living below the 1000-year storm surge elevation; people who experience flooding by storm surge, including the influence of sea defences.	The number of people flooded by storm surge will be more than five times higher due to SLR by the 2080s. Many will experience annual or more frequent flooding requiring some response e.g. (increased protection, migration)	Up to 195 million people might need to respond to frequent flooding by 2080s. Potential responses include migration as well as upgrading and flood protection.
Small *et al* 2000 *(see also LECZ)*	• EROS DEM • GPW2 (1994) • GSHHS shoreline • Tide gauge sea level data • SLR scenarios	Estimation of global population and land area with respect to elevation, proximity to coastline, SLR and coastal hazards.	2000	Population living at low elevations (below 20 m) and near coastlines (within 20 km)	While large numbers of people live at low elevation near the coast, higher resolution population datasets and DEMs are needed to assess risk from coastal hazards and SLR.	Rates of urbanisation will affect size of populations in low coastal areas, particularly in countries with major cities near coasts.
Nicholls and Lowe 2004	• Four scenarios, as per Nicholls 2004 (see above). Additionally, this analysis considers the unmitigated scenario (IS92a).	To consider the potential benefits of mitigation of human-induced climate change in coastal areas, with an emphasis on SLR.	1990, 2080, 2140,	Population in ‘near coastal zone’; within 100 km horizontally and 100 m vertically of coastline.	Under the unmitigated scenario significant impacts above baseline are not apparent until the 2050s. However, after onset, impacts are significant with flooding due to SLR estimated to impact many millions or even hundreds of millions of additional people per year. Adaptive response could be protection measures (dike building) to migration out of flooded areas.	Coastal populations are growing (net coastward migration). Adaptive response to SLR could include migration out of flooded areas.
Anthoff *et al* 2006 *(see also LECZ)* (Working Paper)	• GLOBE DEM • GPW3 population • Tidal range data • GDP/capita: World Resources Institute	Estimation of damages due to SLR scenarios of 0.5 m, 1 m and 2 m by 2100.	2100	Population living within 0.5 m, 1 m and 2 m of sea-level	Damage cost of SLR include: dryland lost, wetland lost, building protection against SLR, the costs of displaced people.	The number of forced migrants due to SLR is a function of population density and area of dry land lost. With no protection, the costs of SLR increase due to land loss and displacement.
Rowley *et al* 2007	• GLOBE DEM • ETOP02 DEM • LandScan population	‘Inundation’ approach to determine land area lost and current population affected by hypothetical sea level increases of between 1 and 6 meters.	Future levels of SLR increase	Population living in inundation zones with sea level rise (for increments of 1–6 m)	Inundated areas ranged between 1.1–2.2 million km^2^ (for 1–6 m of SLR) and affected population ranged from 107.94 to 431.44 million, respectively. Further analysis is available at regional scale for parts of the world (e.g. SE Asia, NW Europe).	SLR will cause inundation of coastal land and the resulting displacement of millions of coastal residents. With 6 m of SLR, for example, 431 million coastal residents would be affected.
Nicholls *et al* 2008b	• GPW3 population • LandScan 2003 • SRTM30 DEM • Land use data, IMAGE (2002) • Land Ocean Interactions in the Coastal Zone typology dataset (tidal range data)	Global implications of abrupt SLR of 5 m, triggered by a hypothetical collapse of the West Antarctic Ice Sheet, including population displacement both with and without coastal defence.	2000 population data, but with a future-oriented scenario.	Global exposure of population, as a function of 1 m and 5 m SLR, calculated relative to high water.	Based on 2000 data, 131 million people would be exposed to SLR of 1 m with 2.5 million km^2^ of land area inundated. 410 million people would be exposed to 5 m SLR with 4.1 million km^2^ of land area inundated.	Land loss is assumed to lead to forced migration, under SLR scenarios. Without WAIS collapse, displacement starts at 75 000 people per year, but falls to 5000 people in 2050 as defence standards improve. With WAIS collapse in 100 years, forced migrants peak at 350 000 a year, with 15 million displaced by an extreme collapse scenario (2030 to 2130) even if most coastal populations are protected.
Li *et al* 2009	• ETOPO5 DEM • ETOPO2 DEM • GLOBE DEM • GTOPO30 DEM • SRTM DEM • Landscan population dataset (2004) • UMD landcover dataset	To assess and visualize the global impacts of potential inundation based on hypothetical global sea level increases of one to six meters.	Impact of SLR of 1, 2, 3, 4, 5 and 6 m for current population.	Population living within a ‘potentially inundated area’ under different SLR scenarios.	Population at risk due to potential inundation ranges from 107.9 million people with 1 m SLR to 431.4 million with 6 m SLR increment.	Not discussed
Nicholls *et al* 2011	• DIVA model with a focus on the variables of flooding and submergence and erosion (with and without adaptation) • Glacial isostatic adjustment (Peltier 2000a, Peltier 2000b)	Potential SLR by 2100 for a beyond 4 °C scenario, and estimates of SLR impacts, both with and without adaptation.	2100	People displaced by SLR due to land-loss via erosion, submergence and flooding. Flooding threshold return rate for abandonment set at > 1 in 1 year frequency.	SLR by 2100, for a temperature rise of 4 °C or more, is estimated to be between 0.5–2.0 m. Assuming no adaptation, there is the **risk of displacement** of between 72 and 187 million people over the century (0.9–2.4% of global population). With adaptation (e.g. dikes, dune nourishment, the number of people displaced falls to 41 000–305 000 people by 2100.	With SLR of 0.5–2.0 m by 2100 for a 4°C increase, there is the risk of forced displacement of up to 187 million people (2.4% of global population). This is potentially avoidable by protection. The models assume no coastward migration.
Marzeion and Levermann 2014	• SRTM DEM • ETOPO1 data (for high latitudes) • Glacial isostatic adjustment (Peltier 2000a, 200b) • GRUMP v1 population model	Estimate of loss of land surface, population exposed, and loss of cultural heritage sites due to SLR, for different temperature levels.	Future degrees of warming	Population exposed to GMSLR of 2.3 m per degree of global mean temperature increase.	% of current population impacted by SLR with different levels of warming: 1 °C: 2.2 (1.3–3.9) 2 °C: 4.7 (3.6–7.2) 3 °C: 6.9 (5.1–9.0) 4 °C: 9.1 (7.9–10.8) 5 °C: 10.5 (8.8–11.6)	Not discussed
Brown *et al* 2016	DIVA model with input datasets e.g.: • GLOBE DEM • CIESIN population density (as per 1995) • Isostatic adjustment (Peltier 2000a, Peltier 2000b) • Socioeconomic scenarios	To estimate distribution of coastal impacts, including number of people flooded per year, based on nine scenarios with changes due to SLR and socio-economic conditions.	2000–2100	Population living in the coastal flood hazard zone (below 1:1000 year surge level) and the water level exceedance curve, including the effect of adaptive measures (e.g. dikes)	By the 2090s, a maximum of 134 million people are projected to be flooded annually. But regional scale models of SLR provide improved projections; GMSLR is a poor representation.	Coastward migration of populations cannot be fully accounted for in models due to lack of reliable, consistent data.
Kummu *et al* 2016	Other datasets (e.g. climate, urban population, agriculture, economic, human footprint) • GTOPO30 DEM • Shore line (1:250 000) • Population density, 1900–2005 (HYDE) • Future population density, 2005–2050, IIASA	To examine global population distribution with reference to both elevation above sea level and horizontal proximity to the coast.	1900 to present, 2030, 2050 (where data allow)	Population living below 5 m elevation above sea-level.	The number of people living in zones that are less than 5 m above sea level is calculated to be 290 million (5.4%) in 1990, 380 million (5.6%) in 2010, and 460 million (5.5%) in 2030, and 495 million by 2050.	Not discussed
**Estimates of population living in coastal floodplains or storm surge zones**						
Hoozemans *et al* 1993 (Book)	The database containing: area of coastal flood plain after SLR; the flood exceedance curve for storm surges; average coastal population density in 1990; subsidence; standard of coastal protection.	Estimated flood risk, costs, loss of coastal wetlands, and changes in rice production, assuming 1 m GMSLR.	1990	Population living in 1-in-1000 years storm surge zone (i.e. coastal flood plain)	About 200–250 million people were estimated to live within the 1-in-1000-year coastal flood plain in 1990; 1-meter GMSLR would increase exposure by 50%, assuming no other changes.	Not discussed.
Nicholls 2002	• Hoozemans *et al* (1993) database of key parameters: e.g. coastal flood plain after 3 m SLR, average coastal population density • Per capita GDP as an ‘ability to pay’ for flood protection parameter	To assess the extent to which global SLR exacerbates coastal flood problems.	1990 2020s 2050s 2080s 2100	People in the hazard zone (PHZ) exposed to flooding by 1000-year storm surges ignoring sea defences; the average annual number of people exposed to flooding by storm surge.	10 million people experienced flooding annually in 1990. In 2100, the estimated people in the hazard zone is between 424–755, and the average annual people flooded is 83–510 million people per year and 9–337 million people per year under evolving protection.	Where flooding by storm surge is frequent (i.e. more than once per year) a significant response is expected: upgrade flood protection, migrate, etc.
Nicholls 2004	Four scenarios—with different political, economic, technical social development—are quantified, derived from IPCC Special Report on Emissions Scenarios (SRES), e.g.: GDP and population scenarios, via IPCC Data Distribution Centre (DDC); GMSLR scenarios; subsidence scenarios.	Considers range of GMSLR and socio-economic scenarios on: (1) changes in flooding by storm surges; and (2) potential losses of coastal wetlands through the 21st century.	2020, 2050, 2080	People living below the 1 in 1000-year storm surge elevation (ignoring sea defences). People who experience flooding by storm surge per year, (including the benefits of sea defences).	SLR increases flood impacts although significant impacts are not apparent until the 2080s where between 2 and 50 million additional people are estimated to be flooded under different emissions scenarios (i.e. 7–10 million, 29–50 million, 2–3 million, 16–27 million people/year under the four scenarios respectively).	The number of people affected by flooding will increase due to growing coastal populations, including net coastward migration.
Nicholls and Tol 2006	• SRES population and GDP scenarios from the IPCC Data Distribution Centre	To consider the potential impacts—e.g. economic impacts—of SLR through the twenty-first century, taking account of different climate and socio-economic scenarios.	2020s 2050s 2080s	People living below the 1000-year storm surge elevation (i.e. ignoring dikes). People who experience flooding by storm surge (including effects of dikes).	For all SRES scenarios, the number of people in the coastal flood plain increases by 2050, from a 1990 baseline of 200 million, and then diverges from the 2080s. While climate stabilization reduces impacts, adaptation to SLR is still required.	Not discussed
Pardaens *et al* 2011	• DIVA: SLR projections and integrated socio-biophysical‐economic model of coastal systems • Met Office ocean-atmosphere models: HadCM3C; HadGEM2‐AO	To consider the effect of GHG mitigation policies on 21st century SLR relative to business-as-usual scenario	2020s, 2050s, 2090s, 2100	People flooded globally per year due to SLR and related coastal impacts, assuming no upgrade in defences.	By 2100, without upgrade in defences, around 55% of the 84 million additional people flooded per year due to SLR under business as usual scenario could be avoided under a mitigation scenario which stabilises temperature at a 2-degree increase.	Not discussed
Jongman *et al* 2012	• GADM administrative boundaries • Food Producing Units • PREVIEW: river flood extent • DIVA: coastal flood extents • SRTM & GTOPO30 DEM • HYDE: population; land-use • Penn World tables; GDP per capita	To estimate global economic and population exposure to both river and coastal flooding	1970–2050	Populations exposed to 1-in-100 year coastal and/or river flood events.	Global population exposed to 1/100 year floods reached 271 million in 2010. In the year 2050, an estimated 345 million people will be living in the 1/100 coastal flood areas. Between 1970–2010, an additional 4.7% of the world’s population was exposed to coastal flooding.	Not discussed
Hinkel *et al* 2014	DIVA model with input datasets: • GLOBE DEM dataset • SRTM DEM dataset • GRUMP population dataset v1 • LandScan population dataset	Coastal flood damage and adaptation costs under 21st century SLR are assessed taking account of uncertainties in topography data, population data, protection strategies, socio-economic development and SLR.	2010, 2100	Population living below the 1 in 100-year flood event; population exposed to annual flooding due to SLR.	Without adaptation, 0.2–4.6% of global population is expected to be flooded annually in 2100 under 25–123 cm of GMSLR. In 2010, the population living below the 1 in 100-y flood event plain is estimated to be between 93–310 million, depending on population and DEM datasets.	For many locations, coastal populations are growing due to coastward migration and urbanization, thereby increasing population exposure.
Neumann *et al* 2015 *(also cited in LECZ section)*	• Population estimates for 2000, 2030, 2060 • SRTM30 Enhanced Global Map • MODIS 500 m Map of Global Urban Extent	Scenario-driven projections of impact of SLR on coastal populations by the years 2030 and 2060, with a 2000 baseline. Four population growth scenarios are modelled, that take account of urban and non-urban populations.	2000, 2030, 2060	Population in LECZs and 1-in-100-year coastal floods zones.	The population living in the 100-year flood plain was estimated as follows: 2000 (189.2 million people); 2030 (282.2–285.9 million people); 2060, (315.5–411.3) million people.	Not discussed
Muis *et al* 2016	• Global Tide and Surge Reanalysis (GTSR) dataset • SRTM elevation • GRUMP (2000)	Flood hazard (inundation extent) and flood exposure (exposed people) based on 1 in 100-year extreme sea levels.	2000	Population exposed to 1 in 100-year extreme sea levels, caused by storm surges and high tides, assuming no adaptation.	1.3% of the global population, equal to 76 million people, is living in the 1 in 100-year floodplain (based on population data from year 2000).	Not discussed
Muis *et al* 2017	• Global Tide and Surge Reanalysis (GTSR) dataset • DINAS-COAST Extreme Sea Levels (DCESL) • DIVA • GRUMP	Present-day flood exposure of land area and population below the 1 in 100-year sea levels	2015	Population below the 1 in 100-year sea levels, assuming no flood defences, hydrological connectivity, and planar flood levels	Global exposed population is 28% lower when based on GTSR instead of DCESL. After correcting for vertical data, DCESL estimates 218 million people are exposed and GTSR estimates that 158 million people are exposed.	Without adaptation, risks from coastal flooding are projected to increase further including because of migration towards the coast.
Brown *et al* 2018	DIVA model with input datasets: ALSO • 1-in-100 year flood plain • SRTM DEM • GTOPO30 • GRUMP v1 • Glacial isostatic adjustment • SSPs • SLR scenarios/WASP model	To project land and population exposed in the 1 in 100-year coastal flood plain, for the years 2100 and 2300, taking into account different mitigation and SLR scenarios and SSPs.	2100, 2300	Population in the 1-in-100 year coastal flood plain for different temperature and associated SLR scenarios.	Assuming no population growth after 2100, the proportion of global population exposed to SLR in 2300 is projected to be between 1.5% and 5.4% for the aggressive mitigation and the non-mitigation scenario, respectively. National estimates are available.	Adaptation to coastal flooding required, particularly where large population growth or coastward migration is expected. Populations may not wish to retreat, but in some places this may be the only option.
Kulp and Strauss 2019	• CoastalDEM • SRTM • LandScan (2010 population density data)	Using a new DEM (CoastalDEM), to examine global population living on land below the high tide line currently, mid-century and in 2100. To compare CoastalDEM and SRTM-based values.	2010 2050 2100	People living on land that may be exposed to coastal inundation, either by permanently falling below mean higher high water (MHHW), or temporarily falling below the local annual flood height.	110 M people currently live on land below the high tide line and 250 M on land below annual flood levels. One billion people currently occupy land less than 10 m above current high tide lines. Under high emissions, up to 630 M people live on land below projected annual flood levels for 2100, and up to 340 M for mid-century.	SLR in the US this century may induce large-scale migration away from unprotected coastlines. Global-scale modelling of the timing, locations, and intensity of migratory responses to coastal flooding is needed.
Vafeidis *et al* 2019	• DIVA, including SRTM • Water attenuation rates based on values reported in the literature • One SSP (2) to represent changes in coastal population and assets • SLR projections of 29, 50 and 110 cm by 2100	To explore uncertainty introduced in global assessments of coastal flood exposure and risk when not accounting for water-level attenuation due to land-surface characteristics.	2015	People living in the 1-in-100-year floodplain; expected number of people flooded per year based on sea flood heights and their probability of occurrence	There is a reduction of up to 44% in area exposure and even larger reductions in population exposure and expected flood damages when considering water-level attenuation	Not discussed
**Estimates of population living in low-elevation coastal zones (LECZs) or ‘near-coastal’ zones**						
Hinrichsen 1996 (see also Hinrichsen 1990, Hinrichsen 1994, Hinrichsen 1998)	• Methodology not thoroughly documented	Estimations of the coastal population	1990s	Population living within 200–400 km of coastline.	In 1994, 50% of total global population lived within 200 km of coastline, over two-thirds within 400 km of coastline; by 2025 70% would live within 200 km of coastline	Not discussed
Cohen and Small 1998	• Population distribution based on censuses (1979–94 data) from 217 countries • EROS DEM • Defense Mapping Agency terrain elevation data Level 1 (30)	To quantify the global distribution of the human population by elevation	1994	Global population by elevation (m) and by population density (people/km^2^).	As of 1994, an estimated 1.88 billion people (33.5% of world’s population) lived within 100 vertical meters of sea level (the lowest vertical resolution investigated in this study).	Not discussed
Small *et al* (2000) *(see also SLR)*	• EROS DEM • GPW (1994) • GSHHS shoreline • Tide gauge sea level data • SLR scenarios	Estimation of global population and land area with respect to elevation, proximity to coastline, SLR and coastal hazards.	2000	Population living at low elevations (below 20 m) and near coastlines (within 20 km)	400 million people live within 20 m of sea level and 20 km of a coastline.	Rates of urbanisation will affect population size in low coastal areas, particularly in countries with major cities near coasts.
Small and Nicholls 2003	• GTOPO30 DEM • GPW2 (2000) • Night-time imagery of Visible and Near Infrared (VNIR) emissions (e.g. fires) via The Defense Meteorological Satellite Program/Operational Linescan • High resolution coastline data: World Vector Shoreline, CIA World Data Bank II	Estimation of population distribution and land area in the ‘near-coastal zone’.	1990	Human habitation of near coastal zones (i.e. within 100 horizontal kilometres and 100 vertical meters of a coastline).	The population within the near coastal zone for 1990 was estimated at 1.20 billion. The population living below 20 m elevation (and within 20 vertical km of coast) was found to be 450 million people.	Urban growth from demographic momentum and urban migration would increase the number of people potentially exposed to coastal hazards.
Anthoff *et al* 2006 *(see also SLR)* (Working Paper)	• GLOBE DEM • GPW3 population • Tidal range data • GDP/capita: World Resources Institute	Estimation of population living within 1 m and 10 m of mean high water in 1995.	1995	Population living within 1–10 m of high water.	146 million people and 397 million people living within 1 m and 10 m of high water in 1995.	Not discussed (in relation to LECZ). But see SLR above.
Mcgranahan *et al* 2007	• GRUMP urban extent grid (2000) • GRUMP GPW v3 (2004) • SRTM DEM	Distribution of global population in LECZs. This elevation is chosen because estimates based on elevations < 10 m are not reliable.	2000	Population residing in LECZ (i.e. contiguous land area up to 10 m elevation that borders a coastline)	LECZs cover 2% (2.7 million km^2^) of the world’s area and 8% (0.3 million km^2^) of its urban area. It contains 10% (618 million) of the world’s population and 13% (352 million) of its urban population.	Coastal disaster risk reduction will require mitigation, migration and settlement modification. Out migration from LECZs will be important, but costly and disruptive.
Lichter *et al* 2011	• GRUMP population model (2000) • LandScan population (2006) • GTOPO30 DEM • GLOBE DEM • SRTM30	Comparative analysis of land area and population distribution in LECZ and their susceptibility to future SLR, based on three DEM and two population datasets.	2000 (GRUMP dataset); 2006 (LandScan dataset)	Population living below 1, 2, 3, 4, 5, and 10 m (LECZ) elevation above sea-level.	Variations in results are dependent on the input datasets. For example, depending on choice of DEM and population dataset, the estimated population living in LECZs ranges from 557–709 million. These differences indicate that results should be regarded with caution and with reference to methods and datasets used.	Displacement of coastal inhabitants because of coastal flooding is noted as a potential outcome of future SLR.
Vafeidis *et al* 2011(Foresight Report)	• Landscan population (2008) • GRUMP population (2000) • SRTM DEM • SRTM30 Enhanced Global • GTOPO30 DEM • DIVA Database 1-in-100-year storm surge heights • MODIS 500 m Map of Global Urban Extent (15 arc sec; 2009)	Estimation of land area and number of people located in LECZ for 2000, 2030, and 2060, including estimates based on four scenarios (developed by Foresight) that include demographic change and SLR.	2000, 2030, 2060	Population living in LECZs (i.e. contiguous coastal area that is less than 10 m above sea level)	The number of people living in the 1-in-100-year floodplain in 2000 (considering GMSLR only) is 628 million. In 2030 and 2060, estimated numbers of people in the floodplain increase slightly with SLR. The main increase in exposure is the result of demographic changes. Asia has the largest proportion of people living in LECZs, in the base year 2000 and in all future forecasts. Further analysis available at national and regional scale.	Modelling accounts for internal migration to the coast.
Mondal and Tatem 2012	• LandScan (2008 version) • GRUMP (2000 version 1, projected to 2008) • SRTM30 Enhanced Global Map (enhanced with GTOPO30 and ocean bathymetry data from ETOPO2)	Demonstrate the variability in estimates of LECZ population size based on use of different population datasets, namely LandScan and GRUMP.	2008	Population residing in LECZ (i.e. contiguous land area up to 10 m elevation that borders a coastline)	Estimates of proportions of national populations in LECZ vary by between 0.1% to 45%, depending on the dataset. Choice of dataset can lead to a difference of more than 7.5 million vulnerable people for countries with extensive coastal populations.	Not discussed.
Neumann *et al* 2015 *(see coastal flood plain)*	• Population estimates for 2000, 2030, 2060 • SRTM30 Enhanced Global Map • MODIS 500 m Map of Global Urban Extent	Scenario-driven projections of impact of SLR on coastal populations by the years 2030 and 2060, with a 2000 baseline. Four population growth scenarios are modelled, that take account of urban and non-urban populations.	2000, 2030, 2060	Population in LECZs and 1-in-100-year coastal floods zones.	The population living in LECZs was estimated as follows: 2000 (625.2 million); 2030 (879.1–948.9 million); 2060 (1052.8–1388.2 million).	Not discussed.
Jones and O’Neill 2016	• Shared Socioeconomic Pathways (SSPs) • GPW 2000 • GRUMP population	To identify global-scale population scenarios for the year 2100, including populations living in LECZ, taking account of the SSPs.	2100	Population residing in LECZ (i.e. contiguous land area under 10 m in elevation that borders a coastline).	The population living in LECZs will change from 702 million in the year 2000 to between 493–1146 million in the year 2100, depending on the socioeconomic pathway.	Different SSPs account for human migration patterns; migration as a function of exposure is not discussed.
Merkens *et al* 2016	• GRUMP population dataset • GRUMP urban extent grid • SRTM v4.1 DEM • GTOPO30 DEM (for high latitudes) • IIASA national urban and rural population projection until 2100 • urbanisation rates data (NCAR) • country economic growth rates	Spatial projections of global coastal population distribution for the five basic Shared Socioeconomic Pathways (SSPs).	2000, 2050, 2100	Population living in LECZs (<10 m above sea level).	With 2000 as a baseline, the population living in LECZs will change from 637 million to—depending on the societal scenario chosen—1005–1091 million by 2050 and 830–1184 million by 2100. Asia expects the highest absolute growth and Africa the highest relative growth.	Explicitly includes coastal migration drivers to develop nuanced accounts of coastal populations for different SSPs. These can be used to assess exposure of population to climate-change impacts.

a indicates publications that are not peer reviewed journal articles.

ASLR = accelerated sea-level rise

CIESIN = Center for International Earth Science Information Network

DCESL = DINAS-COAST Extreme Sea Levels

DEM = Digital Elevation Model

DIVA = Dynamic Interactive Vulnerability Assessment

EROS = Earth Resources Observation Systems

ETOPO = Earth Topography Dataset

GDP = Gross Domestic Product

GSHHG = A Global Self-consistent, Hierarchical, High-resolution Geography Database

GMSLR = global mean sea-level rise

GRUMP = Global Rural-Urban Mapping Project

GPW = Gridded Population of the World

GTSR = Global Tide and Surge Reanalysis

IIASA = International Institute for Applied Systems Analysis

IPCC = Intergovernmental Panel on Climate Change

LECZ = low-elevation coastal zone

MODIS = Moderate Resolution Imaging Spectroradiometer

NCAR = National Center for Atmospheric Research

ppmv = parts per million volume

SLR = sea-level rise

SRTM = Shuttle Radar Topography Mission

SSP = shared socioeconomic pathway

UMD = University of Maryland Dataset

WASP = Warming Acidification and Sea-level Projector

## 3. Results: scenarios and numbers for population exposure

### 3.1. Overview

Global and near-global estimates of exposure to SLR and associated hazards variously take into account different emission and socioeconomic scenarios, focus on different timeframes and geographic scales, and employ different datasets and methods of estimating land area and population distributions in coastal zones, from simple inundation models to more complex vulnerability assessments (Lichter *et al* 2011). Shown in table 1, the publications found for this review focus on three categories of exposure, reviewed in three subsections below, based on definitions in the publications: (i) the population impacted by specified levels of SLR; (ii) the number of people living in floodplains that are subject to coastal flood events with a specific return period; and (iii) the population living in LECZs defined as the contiguous and hydrologically connected zone of land along the coast and below 10 m of elevation (Mcgranahan *et al* 2007) or in the ‘near coastal’ zone (i.e. within 100 km of a coast).

The most conservative definition of populations exposed to SLR identifies those living below the future projected sea level with potential for permanent inundation. Residence in the projected floodplain demarcates exposure to SLR-related impacts, but not necessarily permanent inundation. Residence in a LECZ is the broadest definition of exposure to SLR. These three exposure categories are related, can be expressed differently among publications (see table 1), can have ambiguities in their delineation, and are sometimes combined in different ways for estimates and models. For example, DIVA (Dynamic Interactive Vulnerability Assessment) is an integrated research model of coastal systems that assesses climatic and socioeconomic scenarios and considers diverse processes such as SLR, coastal erosion, coastal flooding (including rivers), wetland change, and salinity intrusion into deltas and estuaries (Mcleod *et al* 2010).

### 3.2. Population impacted by specified levels of SLR

Eleven publications provide global estimates of the land area and number of people exposed to specific levels of SLR at different timescales (table 1). For example, Li *et al* (2009) estimate that based on current population distributions, 1 m of SLR would expose 108 million people to potential inundation and up to 431 million people at the 6 m increment. Marzeion and Levermann (2014) assume 2.3 m of GMSLR per degree of global mean temperature increase; they find that between 2.2% and 10.5% of the world’s population would be directly exposed to SLR for 1 °C and 5 °C of temperature increase, respectively. Hinkel *et al* (2014) estimate that 1.2 m of SLR by 2100 would threaten up to 649 million people (4.6% of the world’s population) assuming a global population of 14.1 billion. Brown *et al* (2018) consider climate scenarios of 1.5 °C and 2.0 °C of warming which correlate to different levels of SLR and then estimate the number of people directly exposed to SLR, assuming no adaptation, in the years 2050, 2100, and 2300.

Estimates of the impact of SLR are generally limited to the 21st century (Nicholls *et al* 1999, Nicholls 2004, Pardaens *et al* 2011, Hinkel *et al* 2014), perhaps due to the time horizon of socioeconomic development and planning and the neatness of 2100 as an event horizon (Marzeion and Levermann 2014). But given current emission trajectories, SLR will continue over several centuries (Nicholls and Cazenave 2010, Clark *et al* 2016, Le Cozannet *et al* 2018). Brown *et al* (2018) produce estimates to the year 2300, and project that the proportion of global population exposed to SLR in 2300 is 1.5% for an aggressive mitigation scenario and 5.4% for a non-mitigation scenario (assuming no population growth after 2100).

In each of these publications, the primary point of reference, or ‘independent variable’, is GMSLR. However, estimates are difficult to compare as they focus on different time scales, use different GMSLR values linked to different climate scenarios, and make differing assumptions about population size and distribution. Nonetheless, they convey the scenarios and numbers of people living in places that would be below global mean sea level and permanently inundated, if no other adaptation responses are implemented (which is a big assumption).

### 3.3. Population living in coastal floodplains

Thirteen publications focus on global coastal floodplains based on SLR with particular return periods under present and future scenarios (table 1). Return periods for heights are available via the Global Tides and Surge Reanalysis (GTSR) dataset which provides data on storm surges and extreme sea-levels for the entire world’s coastline and provides estimates of extreme sea levels with a 1-in-100-year return period (see Muis *et al* 2016). Brown *et al*’s (2018) estimates to the year 2300 (section 3.2) apply to the coastal floodplain as well. Neumann *et al* (2015) estimate the number of people living in the 1-in-100-year coastal floodplain at three points in time: 189 million people in 2000, between 283–286 million people in 2030, and between 316–411 million people in 2060.

While these analyses of exposure to coastal floods can inform adaptation planning and policymaking, they are constrained by methodological limitations, computational restrictions, and lack of consistent datasets (M I *et al* 2018. Many studies use the simple ‘bathtub method’ to estimate global impacts of SLR and associated coastal flooding (Lichter *et al* 2011, Hinkel *et al* 2014) in which a pre-determined flood surface is projected onto a digital elevation model (DEM). Yet some studies suggest a pronounced reduction in coastal flood exposure when estimates account for hydrodynamic processes, namely water level attenuation in coastal flood plains due to vegetated surfaces, or the role that wetlands play in offsetting SLR through soil building (Mcleod *et al* 2010, Vafeidis *et al* 2019). Conversely, other local-level studies that combine SLR estimates with wave and erosion models, find there is considerably more land at exposed to flooding or erosion due to SLR than is shown by simple passive flood mapping (Anderson *et al* 2018).

Furthermore, while the concept of return periods—e.g. a 1-in-100-year flood event—is widely used in estimating land area and population exposure to coastal floods, this assumes an unchanging or ‘stationary’ climate in the past and in the future. Given climate change, this is no longer an appropriate way in which to convey coastal flood risk, with SLR and shifts in the frequency and intensity of storms influencing the return periods (Gilleland *et al* 2017). Finally, maximum flood height is only one parameter characterising the potential hazard, as adaptation measures, duration of flooding, and contaminants are also key hazard dimensions. Nonetheless, these estimates highlight that continued climate change is projected to lead to greater exposure of populations to coastal flooding and related impacts such as erosion and saltwater intrusion.

### 3.4. Population living in low-elevation coastal zones (LECZs)

Twelve publications provide global estimates of the number of people living in LECZs, as an indicator of exposure to SLR and coastal flooding (table 1). Many large cities (e.g. Shanghai, Kolkata, Jakarta, London, and New York City) are situated at least partially within LECZs. Mcgranahan *et al* (2007) find that 618 million people were living in LECZs in 2000, while Lichter *et al* (2011) estimate that the population living in LECZs ranges from 557.1 million to 709.1 million. Variations in estimates are determined largely by dataset choice. Studies have also projected future population numbers living in LECZs. They find that by 2100, LECZs population could be as low as 492.7 million in a world of increasing socioeconomic inequality with low-income growth (Jones and O’Neill 2016) and as high as 1.2 billion in the highest economic growth scenarios (Jones and O’Neill 2016, Merkens *et al* 2016). Other studies have estimated that, with high population growth, as many as 1.4 billion people could inhabit LECZs by as early as 2060 (Mcgranahan *et al* 2007, Vafeidis *et al* 2011, Neumann *et al* 2015).

While Mcgranahan *et al* (2007) state that LECZs are a defensible measure of exposure to SLR because elevation data below 10 m are not reliable, others warn that given the uncertainties inherent in elevation and population datasets, any LECZ estimates should be regarded with caution and with reference to methods and datasets used (Lichter *et al* 2011, Mondal and Tatem 2012). Living in a LECZ does not necessarily entail current or future exposure to SLR-related hazards, as flooding is not a certainty and because of the wide variety of potential adaptation measures (Yamamoto and Esteban 2014), but estimates draw attention to the concentration of populations and physical assets in LECZs.

## 4. Challenges of measuring population exposure to SLR

This review found numerous challenges in the literature when measuring population exposure to SLR and related impacts. The estimates are based on gridded datasets that include DEMs, flooding and extreme sea-levels, and population distribution. Hinkel *et al* (2014), Lichter *et al* (2011), and Mondal and Tatem (2012) have shown that estimates of land and population exposure to SLR and coastal flooding vary significantly according to which datasets are employed. Final estimates depend on the input data, and decisions about key parameters such as time horizons, warming scenarios, and ecological or socioeconomic processes and feedbacks including adaptation measures assumed. Four main challenges are discussed here based on our review and analysis.

First, estimates of populations exposed to SLR rely on elevation data to define zones of inundation or potential hazard parameters (Small and Nicholls 2003, Ericson *et al* 2006, Mcgranahan *et al* 2007, Lichter *et al* 2011). Global DEM datasets include GLOBE which combines six gridded DEMs and five cartographic sources; the US Geological Survey GTOPO30 which combines eight raster and vector sources of topographic information; and the Shuttle Radar Topography Mission (SRTM) elevation data with a vertical resolution of 1 m and spatial resolution of approximately 90 m at the equator (Mcgranahan *et al* 2007, Lichter *et al* 2011, Brown *et al* 2018). Any DEM has vertical and horizontal uncertainties (Wolff *et al* 2016). For example, while there are enhanced versions of SRTM data (see Mondal and Tatem 2012, Kulp and Strauss 2019), SRTM datasets have uncertainties in urban and forested areas where radar technologies capture infrastructure or tree elevation as opposed to ground elevation (Dasgupta *et al* 2011, Marzeion and Levermann 2014). Global mean error in SRTM’s 1–20 m elevation band has been found to be 1.9 m (and 3.7 m in the US) (Kulp and Strauss 2019). Choice of DEM also has significant effects on estimates. Hinkel *et al* (2014) found the estimated number of people flooded according to the GLOBE elevation model to be double that calculated using the SRTM elevation model, and Kulp and Strauss (2019) found that using CoastalDEM instead of SRTM resulted in estimates of population exposure to extreme coastal water level that were three or more times higher. Improvements in elevation datasets are required to enable accurate estimates of land area exposure (Gesch 2009).

Second, estimating coastal floodplains and potential coastal flooding requires datasets on extreme sea levels. A significant limitation of flood analysis at all scales is limited availability of accurate datasets (Gesch 2009, Mondal and Tatem 2012, Neumann *et al* 2015). In their analysis of coastal flood exposure, Muis *et al* (2017) found that correcting vertical data of sea-level extremes and land elevation for two sea-level datasets—DINAS-COAST Extreme Sea Levels (DCESL) dataset and the GTSR dataset—resulted in an increase of 16% and 20% respectively in flood exposed land, and 39% and 60% respectively for exposed populations. Moreover, there are other drivers of flooding including storm intensity, and climate change affects frequencies, magnitudes, and tracks of storms thereby yielding low confidence in how storm surges and extreme sea levels may alter over time (Marzeion and Levermann 2014, Brown *et al* 2018, Knutson *et al* 2019). Hazard parameters must be set within models, but it is not always clear which hazard parameters to select, or whether to select extremes or means.

Third, estimates of population exposure to SLR require population distribution datasets. Key datasets are the Gridded Population of the World (GPW), the Global Rural Urban Mapping Project (GRUMP), and LandScan Global Population database, all of which are developed from census data. Census data are available by census accounting units, with uncertainty in the spatial distribution of populations within each unit. GPW was the first global and widely available dataset that transformed census data to a grid; it emphasises input data rather than modelling distributions (Nicholls *et al* 2008a). GRUMP combines population data with census units, allocating people into urban or rural areas to coincide with UN estimates and using an urban extent assessment derived mostly from the night-time lights dataset of the US National Oceanic and Atmospheric Administration (NOAA) (Mcgranahan *et al* 2007, CIESIN, IFPRI, World Bank and CIAT 2011, Mondal and Tatem 2012). LandScan disaggregates census data within administrative boundaries based on weightings derived from land cover data, proximity to roads, slope, and populated areas (Bhaduri *et al* 2007, Mondal and Tatem 2012). As always, all these datasets have limitations. Spatially detailed census data are often not available for low-income countries; some census data are over 10 years old; informal settlements and undocumented people might not be accounted for in census data; and datasets that use night-time lights as a proxy for population can miss smaller coastal settlements with limited development and where electricity supply is intermittent or unavailable (Dugoua *et al* 2017). Different datasets produce differing population distributions. An analysis of variation in estimates of populations in LECZ as derived from LandScan and GRUMP found that eight of the top ten locations with the largest differences in estimates were small low-lying island countries or territories, including for example Tuvalu (Mondal and Tatem 2012). Consequently, the limited spatial resolution of census data means there is uncertainty as to the location of populations relative to SLR and its related hazards (Small and Nicholls 2003, Foley 2018).

Finally, many studies set specified levels of future GMSLR based on different emission and warming pathways over the coming decades, centuries, and millennia (e.g. Pfeffer *et al* 2008, Brown *et al* 2016, Clark *et al* 2016, Mengel *et al* 2016). Debates regarding GMSLR estimates and forecasts relate to spatial variations, temporal uncertainties, rates of ice mass loss especially from Greenland and Antarctica, ocean dynamics, emission scenarios, and changes in gravity associated with water mass redistribution, leading to significant regional variations from the global mean (Clark *et al* 2016, Jevrejeva *et al* 2016, Mengel *et al* 2016, Geisler and Currens 2017). Subsidence and isostatic uplift further affect local sea level projections (Hinkel *et al* 2014, Erkens *et al* 2015, Brown and Nicholls 2015). There are temporal uncertainties in forecasting GMSLR associated with projected rates (e.g. Bindoff *et al* 2007, Solomon *et al* 2007); natural, multi-decadal oscillations (Sérazin *et al* 2016); and the pace of ice mass loss from the Greenland and Antarctica ice sheets (Tol *et al* 2006, Hansen 2007, Pfeffer *et al* 2008, Nicholls *et al* 2011, Jevrejeva *et al* 2016). Thus, future GMSLR is uncertain, something that many studies address by using higher and lower bounds of GMSLR in their analyses (IPCC 2019, c.f. Marzeion and Levermann 2014).

In summary, reliability of the estimates of both current and future population exposure to GMSLR and related hazards depend on the reliability of input datasets, with precision not always reflecting accuracy. Global quantitative estimates rely on global datasets, yet there are widely acknowledged challenges in estimating land elevation, extreme sea levels, population distribution, and GMSLR scenarios. These problems are amplified for studies seeking to estimate population exposure to GMSLR in the future. For example, the uncertainties in current population distribution estimates mean that future estimates also have large uncertainties.

## 5. Relevance of population exposure to SLR for migration

While noting the challenges of estimating population exposure to SLR, most global estimates are in the order of tens or hundreds of millions of people exposed to coastal inundation and coastal flooding for different timeframes and scenarios. Given the current and expected impact of SLR, exposed populations must adapt (Murphy 2015). Adaptation to SLR and related hazards is widely defined under three main categories (Dronkers *et al* 1990, Brown *et al* 2016, IPCC 2019):(1) protection (measures reducing the parameters or likelihood of inundation in a specific location); (2) accommodation (policies or actions to live with the consequences of SLR); and (3) migration or relocation (planned movements of populations, sometimes termed ‘managed retreat’ or ‘managed realignment’). Despite the possibility for different adaptive measures to SLR, migration or relocation—also variously referred to as retreat, displacement, resettlement, realignment, evacuation, and abandonment—has received significant attention in public and policy discussion.

First suggested in the 1970s (e.g. Brown 1976), the term ‘environmental refugee’ was the focus of much debate before being largely discredited on the basis of its legal definition, since the United Nations (UNHCR 1951/1967 ) does not include any environmental basis for claiming or granting refugee status. Additionally, the multi-causal nature of migration (Foresight: Migration and Global Environmental Change 2011, Felli and Castree 2012, Fiddian-Qasmiyeh *et al* 2016, Ayeb-Karlsson *et al* 2018) undermined concepts of environmental, climate, and climate change refugees, along with increasing resistance among residents of small island states to narratives of disappearing islands and population displacement as iconic examples of the threat of climate change (Farbotko 2010). Nonetheless, a recent ruling by the United Nations in response to a protection claim from a resident of Kiribati (OHCHR 2020) referred to ‘*climate change refugees*’ as people where ‘*the risk of serious harm arises from environmental factors indirectly caused by humans, rather than from violent acts*’ (OHCHR 2020, p 8). There are also emerging studies documenting early cases of human migration and relocation linked to the perceived impacts of SLR (c.f. Mcmichael and Katonivualiku 2020). Some countries—e.g. Micronesia, the Marshall Islands, the Solomon Islands, Fiji, and Kiribati—are developing policy and practice responses that include migration and planned resettlement of vulnerable populations in response to perceived climate impacts such as SLR (Hauer *et al* 2020). Despite the discursive, legal, and policy developments that link SLR to human migration, as well as emerging examples of low-lying places where SLR is considered a key climate change-related hazard which could affect migration decision-making, the question remains as to how, where, and to what extent population exposure to SLR might shape human mobility globally.

Twenty of the 33 publications reviewed in this article discuss connections between population migration and SLR. For example, Nicholls *et al* (2011) suggest that 2 m of SLR by 2100 yields the risk of ‘*forced displacement*’ of up to 187 million people. They suggest that, although displacement is avoidable through protection, the likelihood of protection being successfully implemented is lowest in small islands, Africa, and parts of Asia, hence these regions are likely to experience coastal abandonment. Brown *et al* (2018) note that although people may not want to move from their coastal sites, in some places this may be the most viable option, highlighting low-lying settlements in Fiji, the Maldives, and Panama. Kulp and Strauss (2019) suggest that coastal communities must prepare for difficult futures including large-scale migration away from coastlines, and they call for global modelling that accounts for the timing, locations, and scale of human migration in response to worsening coastal flooding. Nicholls *et al* (2008b) consider the scenario of West Antarctic Ice Sheet collapse (WAIS) producing 5 m of SLR, and consequent land loss that would lead to forced migration. Rowley *et al* (2007) suggest that inundation of coastal land due to SLR could displace millions of coastal residents, with up to 430 million people affected by SLR of 6 m.

Other studies that focus on wider SLR-related hazards such as coastal flooding have also suggested migration will be a key response. For example, Hinkel *et al* (2014) argue that where protection is not in place, populations will move in response to growing flood risk. Mcgranahan *et al* (2007) note that migration represents a key risk reduction strategy for populations in coastal lowlands, albeit a costly and disruptive form of adaptation. Given that exposure to SLR is not a reliable proxy indicator for migration, none of these studies rigorously or reliably quantifies the number of people who might be expected to move due to SLR or could do so. Beyond estimates of exposure, the connections between SLR and migration are complex and uncertain.

Consequently, population exposure to SLR and related impacts should not be conflated with inevitable migration. As has been widely noted, migration decisions are shaped by more than environmental factors, including economic, social, demographic, institutional, and political dimensions (Nicholls *et al* 2008a, Foresight: Migration and Global Environmental Change 2011, Felli and Castree 2012, Fiddian-Qasmiyeh *et al* 2016, Ayeb-Karlsson *et al* 2018, Hauer *et al* 2020). SLR could generate ‘trapped’ populations who have a desire to move but not the necessary resources, and there will be those who prefer not to move for social, cultural, and political reasons including place attachment (Farbotko and Mcmichael 2019).

Further, migration can be prevented or forestalled through other adaptive strategies. Nicholls and Cazenave (2010) emphasise that humans have historically adapted to coastal change, and many of the world’s most populated coastlines and coastal cities are currently managed and engineered for flood risk (see also Hallegatte *et al* 2013). Over the 20th century, coasts have subsided by up to 5 m in Tokyo, 3 m in Shanghai, and 2 m in Bangkok; these cities depend on flood- and water management infrastructure to prevent further submergence. Some countries and cities with large coastal populations will try to adapt *in situ* (Davis *et al* 2018). Indeed, Lincke and Hinkel (2018) suggested that for 90% of exposed populations, who live on only 13% of the world’s coasts, it will be cost-effective to protect. Nicholls *et al* (2008b) find that even in the scenario of the WAIS collapse, significant lengths of the world’s populated coast could be defended, thereby significantly reducing migration.

Publications (including ten from this review) make the important point that coastal populations are growing due to migration into urban sites which increases population exposure to SLR (c.f. Small and Nicholls 2003, Nicholls 2004, Mcgranahan *et al* 2007, Hinkel *et al* 2014, Neumann *et al* 2015, Kummu *et al* 2016, Muis *et al* 2017). Jones and O’Neill (2016) suggest that models of future population distribution do not adequately account for the potential impacts of climate change. They argue that population distribution may be shaped by migration away from inland drought-affected regions and into coastal areas, and that population projections should include these parameters. These considerations highlight that populations are not static unless affected by SLR and related impacts. As the migration and mobilities literature has long explained (e.g. Fiddian-Qasmiyeh *et al* 2016), people continually move into and away from sites for numerous reasons, including coasts, so any estimates are subject to large uncertainties. These uncertainties do not decrease due to exposure to SLR.

Studies with a tighter geographic focus address some of these challenges, as they draw on datasets with higher spatial resolution and can include parameters that address the specificities of local social, economic, demographic, and political contexts (c.f. Hauer 2017, Chen and Mueller 2018). For example, Davis *et al* (2018) modify a diffusion-based model of human mobility in combination with population, elevation, and climatic data to forecast SLR-driven migration in Bangladesh in the years 2050 and 2100. They predict that 0.9 million people by 2050 and 2.1 million people by 2100 could be displaced by permanent inundation, predominantly within the southern half of Bangladesh. Hauer (2017) focuses on populations in the US, estimating both the number and destinations of potential SLR migrants by 2100. The analysis uses county-level projected population data, county-to-county migration flow data, and household economic status while assuming a 1.8 m SLR scenario, in order to simulate expected impacts. Hauer (2017) finds that by 2100, every US state and 56% of counties could be affected by SLR-related migration, of people moving either in or out.

Another advantage of more localised studies is that they can account for other environmental changes and coastal geomorphology that interact with SLR such as pre-existing subsidence, coastal erosion due to ecosystem destruction (e.g. wetlands, mangroves, and coral reefs), land use changes, or groundwater extraction (Mcleod *et al* 2010, Arkema *et al* 2013). Here, SLR and associated coastal change, might be a necessary but not sufficient driver of migration. For example, research in Louisiana illustrates that 5000 km^2^ of coastal land has been lost since 1932—due to local, regional, and global factors driving relative sea level change—yet Louisiana’s population has not moved landward in concert with observed shoreline encroachment (Hauer *et al* 2019). While this article is specifically examining global or near-global estimates for considering the implications of SLR and associated impacts, such as potential migration, more localised studies might be more appropriate.

Nonetheless, data are not readily available in many places identified as vulnerable to the impacts of SLR. For example, in 2014, residents of the small coastal village of Vunidogoloa, Fiji relocated from the foreshore to a higher site within their customary *mataqali* (i.e. clan) land. Over recent years and decades, villagers experienced high tides that regularly flooded their homes and village land, coastal erosion which led to the loss of some homes, storm surges, and saltwater intrusion. Earlier efforts to adapt included ad hoc relocation of houses and construction of two seawalls. The entire village relocated in January 2014, with the support of the Government of Fiji and donor organisations as well as significant community input and resources. Villagers consider their relocation to be driven by climate change, specifically SLR impacts (Mcmichael and Katonivualiku 2020). It is referred to and labelled by the Government of Fiji and institutions and donor organisations as a climate change-related relocation (c.f. The Fijian Government 2019).

Yet, many coastal sites are not well-represented in global gridded population and elevation datasets. An assessment of Pacific island countries and territories found that LandScan tended to overestimate numbers of Fijians in urban centres and GPWv4 tended to over-disperse the population, resulting in a smoothed population distribution rather than small dispersed settlements and urban centres (Andrew *et al* 2019). Moreover, SRTM elevation data could be unreliable in Fiji since SRTM imagery has large errors for sites vegetated with mangroves and evergreen tree cover (Hawker *et al* 2018). Furthermore, there is limited coastal monitoring in Fiji and other Pacific island countries to determine the extent to which coastal erosion and flooding are attributable to climate change related SLR, as opposed to other coastal geomorphology processes associated with subsidence, or human activity such as river dredging and removal of mangroves (Singh *et al* 2019). Vunidogoloa is an example of planned relocation in which emerging, local, coastal exposure aligns with the types of impacts expected with SLR, yet the resolution of global and regional SLR exposure estimates is too coarse to generate meaningful assessment of the causes of local coastal changes.

## 6. Conclusions

Coastal zones are some of the most densely populated areas in the world and include many of the world’s large cities and many of the fastest growing urban areas (Smith 2011, Brown *et al* 2018). There are also coastal zones with small settlements and villages, such as those in outer islands of small island states or rural areas of Belize and Bangladesh. This review shows that the existing estimates of population exposure to GMSLR and related hazards largely depend on the reliability of input datasets and the methodologies adopted. The underlying uncertainties in the elevation datasets continue to result in significant differences in the estimates. Another limitation in the literature arises from the spatially gridded population datasets that are still unable to produce reliable high-resolution estimates in the lowest-lying coastal areas. The uncertainties in the current population distribution likely mean that future estimates will have large uncertainties. Low statistical confidence levels associated with extreme sea levels datasets are also common, especially regarding the vertical data of sea level extremes and land elevation. Finally, despite the fact that future estimates of GMSLR are uncertain, many existing studies do not provide confidence limits of their estimates of population exposure.

Irrespective of the uncertainties, the literature converges on the point that coming decades and centuries will see SLR radically redefine the world’s coastlines (Taherkhani *et al* 2020). Global and near-global population estimates with their ranges highlight the large numbers of people exposed to SLR, both now and in the future. Even with climate change mitigation and more stable global temperatures, the land area and population exposed to SLR seem likely to continue increasing for centuries (Nicholls and Lowe 2004, Hauer *et al* 2020), so climate change adaptation is still required of which population mobility is frequently highlighted.

SLR has the potential to shape population mobility at all scales (Hauer *et al* 2020). There are significant and long-term implications of SLR-related hazards for coastal habitation (Brown *et al* 2018). Migration and relocation are ways for people to adapt to environmental and climatic changes (Black *et al* 2011, Murphy 2015), and migration has been a way of responding to shifting coastlines throughout human history (Fiddian-Qasmiyeh *et al* 2016). The main difference today is that climate and coastal changes contributing to human migration are increasingly human-induced, so global and national policy and practice debates are examining this topic.

Whether or not improved global estimates of population exposure to SLR are needed for policy and decision-making is debatable. Global estimates give a sense of the scale and distribution of exposure, but many other factors are needed for understanding and managing potential impacts and for planning for adaptation locally. Paramount are the moral, ethical, and legal debates regarding population stabilisation, land ownership, resources available, and decision-making for moving or staying. This review’s value in these discussions is demonstrating that (i) despite the uncertainties and unknowns, it is clear that coastal changes and consequences for populations will be large and require significant action, (ii) there is time now to consider thoroughly the situations and options, so this should be done rather than waiting for more urgency, and (iii) many aspects of the topic need to be decoupled to avoid repeating myths or reproducing discursive narratives that uphold global power relations, especially those covering ‘climate refugees’ (Kelman 2019, Ayeb-Karlsson 2020).

For example, this review shows that the (imprecise) global estimates of population exposure to SLR are inaccurate proxies for estimates of SLR-related migration. Migration decisions are shaped by more than exposure to environmental changes (Fiddian-Qasmiyeh *et al* 2016); people may be trapped or choose to remain in sites of high exposure; people move into sites of exposure; and migration can be prevented or delayed through other adaptation and accommodation measures. It is necessary to connect SLR-related hazards to human migration at the temporal and spatial scale of lived experience and decision-making. More work is needed to better understand how to ensure that policies and decision-making focus on the research findings rather than the rhetoric, especially regarding how SLR interacts with other factors to shape migration decision-making.

A key area for further research could be to understand the ways in which adaptation measures (e.g. protection, accommodation, or migration) to SLR are shaped by wealth, including at the societal, household, and individual level. While some continue to highlight GDP (e.g. Kirezci *et al* 2020), this indicator is known to have such severe limitations that robust and meaningful results for policy and decision-making would be much better obtained by extracting information from micro surveys. Additionally, economic analyses suggest that managed migration might be cost-effective for the majority of the world’s coastlines while protection might be pursued selectively in sites with dense populations and assets (Diaz 2016, Jevrejeva *et al* 2018). To illustrate, Hauer (2017) assumes that wealthier households in the US (earning more than US$100 000 per year) are more likely to adapt to SLR *in situ*, and thus unlikely to migrate. Economic analyses cannot fully account for intangible costs, such as health impacts, disrupted place attachments, and loss of culture and wellbeing, so further work should do so.

This work should also include focusing on improving terrain elevations in urban and coastal settings. Furthermore, although this review covers a global phenomenon, more localised studies are needed to better examine locally driven environmental stressors and coastal geomorphology. This will require developing coastal area elevation datasets by combining local terrain information with high-resolution satellite data. One other input into this work is considering scenarios of the Greenland and/or Antarctic ice sheets melting rapidly, because then, adapting *in situ* might not be an option for many. Understanding the drivers and any tipping points for SLR-related migration requires more detailed investigation, which then circles back to the decision-making for adaptation. As this review shows, without ice sheets melting, GMSLR might not exceed the maximum sea level since the last ice age (Dickinson 2009) until next century, in the absence of action related to climate change. Ice sheet melting provides substantially worse scenarios. Decisions need to be made over the next century considering these two widely divergent possibilities.

Thus, global and near-global estimates of SLR-related population exposure and their relevance for migration highlight (i) widespread impacts of human-caused climate change with the single phenomenon of GMSLR affecting a significant proportion of the human population and (ii) challenging circumstances for deciding how to act, considering the wide-ranging options from population stabilisation to engineering coastlines to moving away from the current shores. These decisions might be helped by spatial distribution and resolution of global datasets improving significantly to enable more reliable quantitative assessments of population exposure to SLR and related impacts, but decision-making should not wait for them, instead planning now for the various decision pathways. Meanwhile, people in LECZs are increasingly reporting environmental changes that are potentially attributable to SLR. At this local scale, more effort is needed to understand the complex interactions between localised SLR and related hazards, local social contexts and potential strategies regarding demographics, migration, and (im)mobility.

## Data Availability

No new data were created or analysed in this study.
